# Effect of antibiotic gut microbiota disruption on LPS-induced acute lung inflammation

**DOI:** 10.1371/journal.pone.0241748

**Published:** 2020-11-04

**Authors:** Max C. Jacobs, Jacqueline M. Lankelma, Nora S. Wolff, Floor Hugenholtz, Alex F. de Vos, Tom van der Poll, W. Joost Wiersinga

**Affiliations:** 1 Center for Experimental and Molecular Medicine, Amsterdam UMC, Location Academic Medical Center (AMC), Amsterdam Infection & Immunity Institute, University of Amsterdam, Amsterdam, The Netherlands; 2 Division of Infectious Diseases, Department of Medicine, Amsterdam UMC, Location Academic Medical Center (AMC), Amsterdam Infection & Immunity Institute, University of Amsterdam, Amsterdam, The Netherlands; Forschungszentrum Borstel Leibniz-Zentrum fur Medizin und Biowissenschaften, GERMANY

## Abstract

**Background:**

An increasing body of evidence is indicating that the gut microbiota modulates pulmonary inflammatory responses. This so-called gut–lung axis might be of importance in a whole spectrum of inflammatory pulmonary diseases such as acute respiratory distress syndrome, chronic obstructive pulmonary disease and pneumonia. Here, we investigate the effect of antibiotic disruption of gut microbiota on immune responses in the lung after a intranasal challenge with lipopolysaccharide (LPS).

**Methods/results:**

C57Bl/6 mice were treated for two weeks with broad-spectrum antibiotics supplemented to their drinking water. Afterwards, mice and untreated control mice were inoculated intranasally with LPS. Mice were sacrificed 2 and 6 hours post-challenge, after which bronchoalveolar lavage fluid (BALF) and lung tissues were taken. Gut microbiota analysis showed that antibiotic-treated mice had a pronounced reduction in numbers and diversity of bacteria. A modest, but time consistent, significant increase of interleukin (IL)-6 release was seen in BALF of antibiotic treated mice. Release of tumor necrosis factor alpha (TNFα), however, was not statistically different between groups.

**Conclusion:**

Antibiotic induced microbiota disruption is associated with alterations in host responses during LPS-induced lung inflammation. Further studies are required to determine the clinical relevance of the gut-lung axis in pulmonary infection and inflammation.

## Introduction

Over the past few years, there has been a growing awareness that the gut microbiota could be an important modulator of pulmonary inflammatory diseases [1–3]. This so called gut-lung axis appears to play an active role in pathogenic processes underlying chronic obstructive pulmonary disease, asthma, acute respiratory distress syndrome and pneumonia [4–9]. Generally, a diverse microbiota has been associated with protection from lung inflammation, whereas low intestinal microbial diversity and antibiotic use are associated with incidence and course of pulmonary disease [10].

Lipopolysaccharide (LPS), a major constituent of the outer membrane of gram-negative bacteria, is recognized by Toll-like receptor (TLR)-4 which plays a key role in initiation of the pulmonary inflammatory response. LPS-induced acute lung injury (ALI) in mice is an often used and suitable preclinical model to investigate pathophysiological features of pulmonary inflammation, applicable to multiple diseases [11, 12]. The response to sterile inflammation after gut microbiota depletion has been studied recently in mice [13]. This study it showed that disruption of the microbiota prior to mechanical ventilation resulted in aggravated inflammatory responses and an increased susceptibility to ventilator induced lung injury (VILI). However, to the best of our knowledge the role of the gut microbiota in the host response during LPS-induced lung inflammation has never been studied. Specifically, we set out to investigate whether disrupted gut microbiota affects the dynamics of key cytokines in lung immunity. The aim of the present study was therefore to investigate the effect of antibiotic induced intestinal microbiota disruption on response to LPS induced acute lung inflammation.

## Materials and methods

### Ethics statement

The Animal Care and Use committee of the University of Amsterdam approved all animal experiments. Experiments have been conducted according to national law and guidelines.

### Mice

Mice were used in this study. Mice, i.e. female and specific pathogen-free C57BL/6 wild type mice (Charles River, Maastricht, Netherlands) arrived at our facility at 7–8 weeks of age. At arrival, these were distributed randomly over cages (4 mice per cage) and allowed to acclimatize for 1 week. Subsequently, random cages were assigned to either antibiotics or normal drinking water (2 cages per condition; n = 8 mice per condition unless specified otherwise).

### Antibiotic regimen

Broad-spectrum antibiotics (ampicillin 1.0 g/L; neomycin 1.0 g/L, both Sigma, Zwijndrecht, Netherlands; metronidazole 1.0 g/L, Sanofi-Aventis, Gouda, Netherlands and vancomycin 0.5 g/L, Xellia pharmaceuticals, Copenhagen, Denmark) were supplemented to the mice drinking water for 14 days.

### Microbiota analysis

Repeated bead-beating of the fecal pellets was performed as described elsewhere [14] but with STAR (Stool transport and recovery) buffer (Roche, Basel Switzerland). Following centrifugation, 250 μl supernatant was used with the Maxwell® RSC Blood DNA Kit (Promega, Madison, USA), and the DNA was eluted in 50 μl DNAse free water. Twenty nanograms of DNA were used for the amplification of the V3-V4 region of the 16S rRNA gene as described [15] with barcoded 341 forward and 805 reverse primers for 25 cycles.

For the purification of the amplified product, the AMPure XP beads (Beckman Coulter, Indianapolis, USA) were used according to manufacturer’s guidelines on a Beckman Coulter Biomex FX. The purified product was equimolar mixed and loaded for sequencing on the Illumina MiSeq with the MiSeq V3–600 cycle kit, as instructed by Illumina.

The sequence reads were analysed as follows. Read pairs with perfect matching forward and reverse barcodes were assigned to their corresponding samples.

The forwards and reverse reads were length trimmed at 240 and 210 respectively, which were inferred and merged with ASVs using DADA2 V.1.5.2 [16]. The assignment of taxonomy was done using the DADA2 implementation of the RDP classifier [17] and SILVA 16S reference database [18].

Subsequent statistical tests were performed using the phyloseq and microbiome package in R.

### LPS-induced lung inflammation

After a washout period of 24 hour with normal drinking water, antibiotic treated and control mice were given ultrapure LPS from *Klebsiella pneumoniae* L4268 (Sigma, Zwijndrecht, Netherlands) or *Escherichia coli* O111:B4 (Invivogen, San Diego, CA), diluted in doses of 1 μg or 10 μg in 50 μL sterile pyrogen-free 0.9% saline and instilled intranasally during anaesthesia by inhalation of isoflurane (Abbott Laboratories, Kent, UK). Two or six hours after LPS inoculations, mice were sacrificed as described before [3, 4, 11].

### Assays

Whole-blood and BALF was obtained post-mortem as described before [11]. Cells in BALF were spun down and counted using a Coulter Counter (Beckman Coulter, Woerden, Netherlands). Blood plasma was acquired by spinning down whole-blood and pipetting off the transparent top layer fraction. Cytokines of interest were measured by cytometric bead array (BD Bioscience, San Jose, CA). Myeloperoxidase (MPO) was measured in lung homogenate by enzyme linked immunosorbent assay (ELISA) (R&D Systems, Minneapolis, MN). BALF protein content was measured using a colorimetric bicinchoninic acid (BCA)-assay (ThermoFisher, Bleiswijk, Netherlands).

### Statistical analysis

Data were analysed using unpaired T test, Mann Whitney U test or one-way ANOVA with post-hoc Tukey’s test, when appropriate (GraphPad Prism 8, San Diego, CA). P-values < 0.05 were considered statistically significant.

## Results

**Fig 1:**
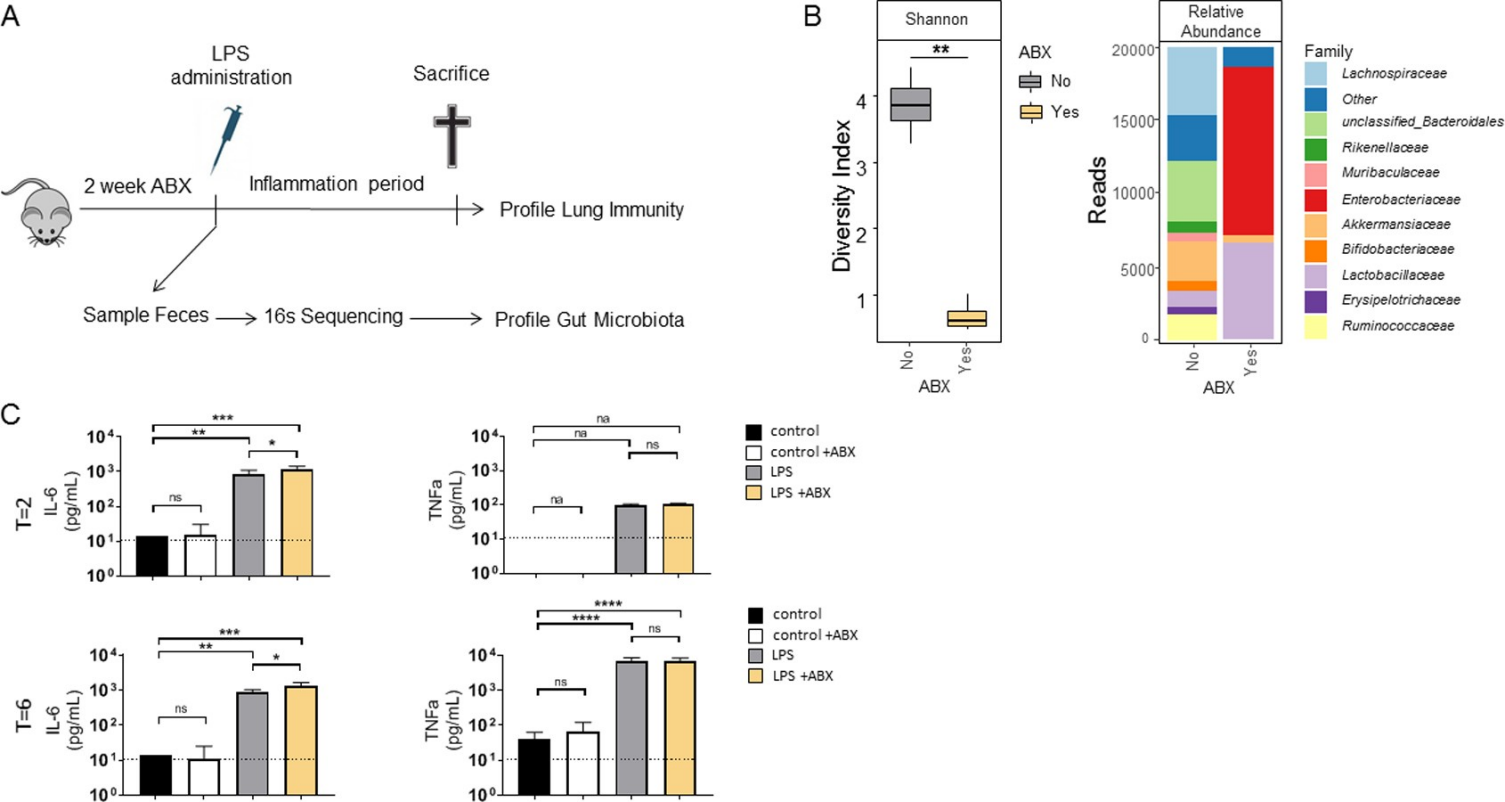
Experimental design, microbiota disruption and pulmonary cytokine production during LPS-induced inflammation in control and antibiotic pre-treated mice. A) Schematic overview of performed experiments. Wildtype C57BL/6 mice were treated with two weeks of antibiotics (ABX: ampicillin, neomycin, metronidazole and vancomycin) in their drinking water after which 1 or 10 μg lipopolysaccharide (LPS) from either *K*. *pneumoniae* or *E*. *coli*, or saline was administered intranasally. Mice were sacrificed 2 or 6 hours after LPS challenge. B) Gut microbiome diversity as measured by Shannon α-diversity of antibiotic (ABX) treated (yellow) and control (grey) mice (left panel). Group averaged relative abundance profiles on Family level between antibiotic treated and control mice (right panel). Both analysis were executed using a rarefaction depth of 20000 reads (n = 12 No ABX, n = 4 Yes ABX mice. Note: The rest of ABX treated mice (n = 12 total) tested did not reach 20000 reads and were therefore excluded from Fig 1B). C) Interleukin (IL)-6 and tumor necrosis factor (TNF)-α were measured in broncho-alveolar lavage fluid of antibiotic pre-treated mice (yellow) or control (grey) sacrificed 2 or 6 hours after intranasal inoculation with 10 μg LPS from *K*. *pneumoniae*. Additional control groups are saline inoculated mice with or without antibiotic pre-treatment (white and black bars, respectively). Dashed line represents detection limit. Data are presented as bar plots showing the mean and standard deviation of the mean (n = 6–8 mice/group). One way ANOVA with post-hoc Tukey’s test. na: not applicable, ns: non-significant; *p<0.05, **p<0.01, ***p<0.001,****P<0.0001.

Antibiotic treated and control mice were given 10 μg LPS from *K*. *pneumoniae*, intranasally (Fig 1A). Miseq sequencing and subsequent analysis using a rarefaction depth of 20000 reads confirmed antibiotic induced microbiota disruption depicted by significant loss in diversity (Fig 1B; left panel) and further illustrated by marked different profiles of Family level relative abundances (Fig 1B; right panel). As expected, LPS administration provokes a significant increase in levels of interleukin (IL)-6 but and tumor necrosis factor (TNF)-α (Fig 1C). In BALF, significantly increased levels of interleukin IL-6 but not of TNF-α was observed in antibiotic pre-treated mice compared to controls, at both 2 and 6 hours post induction (Fig 1C). Analysis of cells BALF revealed no significant differences in total numbers as well as in neutrophil population (Table 1). MPO levels in lung homogenate, a reflection of total number of neutrophils in the lung, was significantly decreased in the antibiotic treated group compared to control mice (Table 1). Furthermore, BALF total protein content, a marker of inflammation and vascular leakage, was not significantly different between groups (Table 1). In blood plasma, we found significantly increased levels of IL-6, TNF-α and Monocyte Chemoattractant Protein 1 (MCP-1) at 2 hours after LPS induction, regardless of ABX pre-treatment (S2 Fig). This effect was not observed at 6 hours after LPS induction. No effect of ABX on protein level increase in blood plasma was observed at either timepoints.

**Table 1:** Cellular influx and protein content of bronchoalveolar lavage fluid during LPS-induced inflammation at t = 6 hours.

	No LPS		LPS	
	Control	ABX treated	Control	ABX treated
**Total cell count (10**^3^**/mL)**	59.4 [51.0–82.5]	34.4 [31.1–54.6]	103.0 [53.3–145.0]	134.7 [118.5–354.6]
**Neutrophils (10**^3^**/mL)**	2.5 [0.9–7.1]	3.0 [1.8–5.3]	43.2 [15.2–72.0]	77.5 [44.8–125.6]
**MPO lung (ng/mL)**	342.8 [302.0–412.1]	322.0 [289.0–369.3]	764.8 [658.8–869.2]	631.2 [575.2–685.2]*
**Protein BALF (μg/mL)**	1255 [1202–1323]	1268 [1159–1341]	1651 [1532–2155]	1596 [1420–3659]

Data are presented as median [interquartile range], Mann Whitney U test: n = 6–8 mice per group.

*p<0.05 compared to control group.

We repeated these experiments using 10 μg LPS from *E*. *coli*. A similar trend of increased TNF-α and significantly increased IL-6 levels at t = 6 in BALF was observed (S1A and S1B Fig) Again, pulmonary neutrophil recruitment was not different in control and antibiotic pre-treated mice (S1 Table). Finally, in order to evaluate dose dependency, we repeated this experiment with 1 μg of LPS. This showed similar results (S1 Fig), with the notion that the increase of TNF-α levels at t = 6 in BALF derived from microbiota disrupted mice reached statistical significance in this experiment (S1B Fig).

## Discussion

To our knowledge, this study is the first to evaluate the effect of antibiotic induced disruption of the intestinal microbiota on LPS-induced lung inflammation. We observed a modest but consistent effect of antibiotic pre-treatment on pulmonary cytokine production after a challenge with LPS from different gram-negative bacteria. No differences in cytokine levels in the systemic compartment were observed. Overall our data suggest that microbiota disruption might predispose to exaggerated inflammatory responses, which was reported before in several mouse models [4, 5, 8, 13]. A healthy, well balanced microbiota might therefore be important for modulating appropriate inflammatory responses. The exact underlying mechanisms remain to be elucidated, however, it is suggested that the gut microbiota is important in the development of healthy innate immunity [19]. Furthermore, antibiotic disruption was found to impair murine haematopoiesis and deteriorate outcome in a pneumonia model [4, 20]. IL-6 in the lungs is produced by both immune and nonimmune cells after various stimuli and can have a wide range of effects. Increased levels of this cytokine have been implicated in various (pulmonary) inflammatory diseases. For instance, IL-6 is increased in BALF and sputum of asthma patients. Besides being present as an immune modulator in various lung diseases, IL-6 has also been suggested to play a role in pathogenesis of asthma and COPD [21]. The observation of decreased levels of MPO in homogenates of antibiotic treated animals lung might indicate immature neutrophils as a result of impaired hematopoiesis. This hypothesis, however, should be addressed in more detail in follow-up studies. Overall, these results are in line with previous reports on altered pulmonary immunity after interventions affecting the intestinal microbiota, in models of pneumonia, allergic airway disease and mechanical ventilation [4, 5, 8, 13].

This study has a number of limitations. Although our antibiotic regimen with known effect on gut microbiota included non-absorbable antibiotics [4], we cannot completely rule out a systemic, potentially immunomodulatory, effect. In the current study, however, we found no significant differences on systemic parameters between antibiotic treated and control mice. Furthermore, we have previously assessed this regimen’s effect on lung microbiota composition, and found no differences between antibiotic treated and control mice [3]. In addition, mice from a different vendor may have a different microbiota and might therefore display different inflammatory responses, as was demonstrated for sepsis as well as malaria severity [22, 23].

In conclusion, antibiotic induced microbiota disruption is associated with alterations in host responses during LPS-induced lung inflammation. An increased pro-inflammatory, IL-6 driven response after treatment with antibiotics could thus contribute to development or severity of pulmonary diseases. Further studies, addressing the effect of gut microbiota depletion on immune cell function, are required to determine the underlying mechanisms as well as the potential clinical relevance of the gut-lung axis in pulmonary infection and inflammation.

## Supporting information

S1 Fig Pulmonary cytokine production 2 and 6 hours after lipopolysaccharide (LPS) induced inflammation in control and antibiotic pre-treated mice. (TIF)

S2 Fig Cytokine production in blood plasma 2 and 6 hour after lipopolysaccharide (LPS) induced lung inflammation in antibiotic treated and control mice. (TIF)

S1 Table Neutrophil influx and protein content of broncho-alveolar lavage fluid during LPS-induced inflammation. (TIF)
