# Supplementary figures and images for: Effect of antibiotic gut microbiota disruption on LPS-induced acute lung inflammation
Source: PLoS One. 2020 Nov 4;15(11):e0241748. doi: 10.1371/journal.pone.0241748 (PMC7641457; doi:10.1371/journal.pone.0241748)

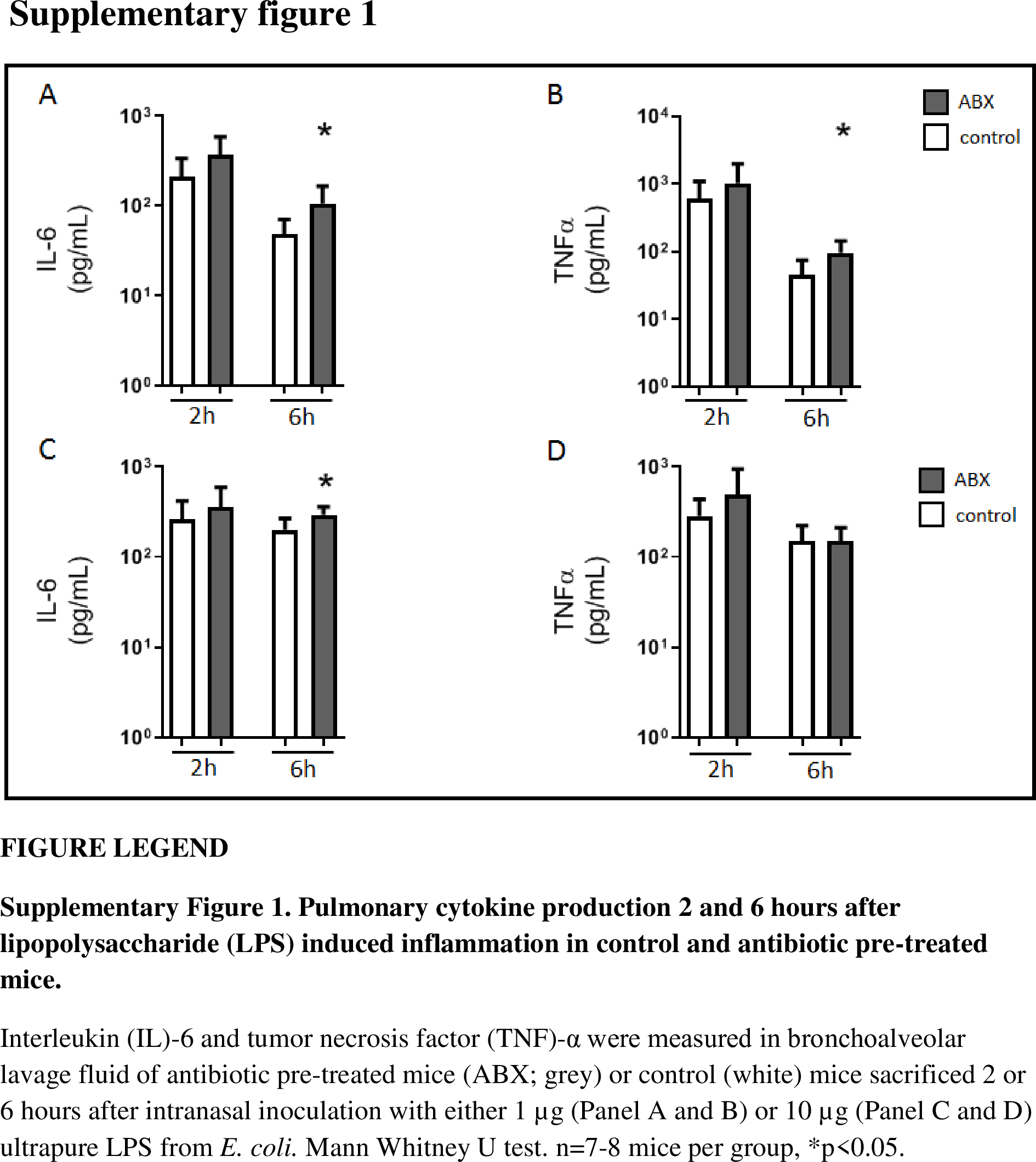

Supplement: S1 Fig — (TIF) [file pone.0241748.s001.tif]

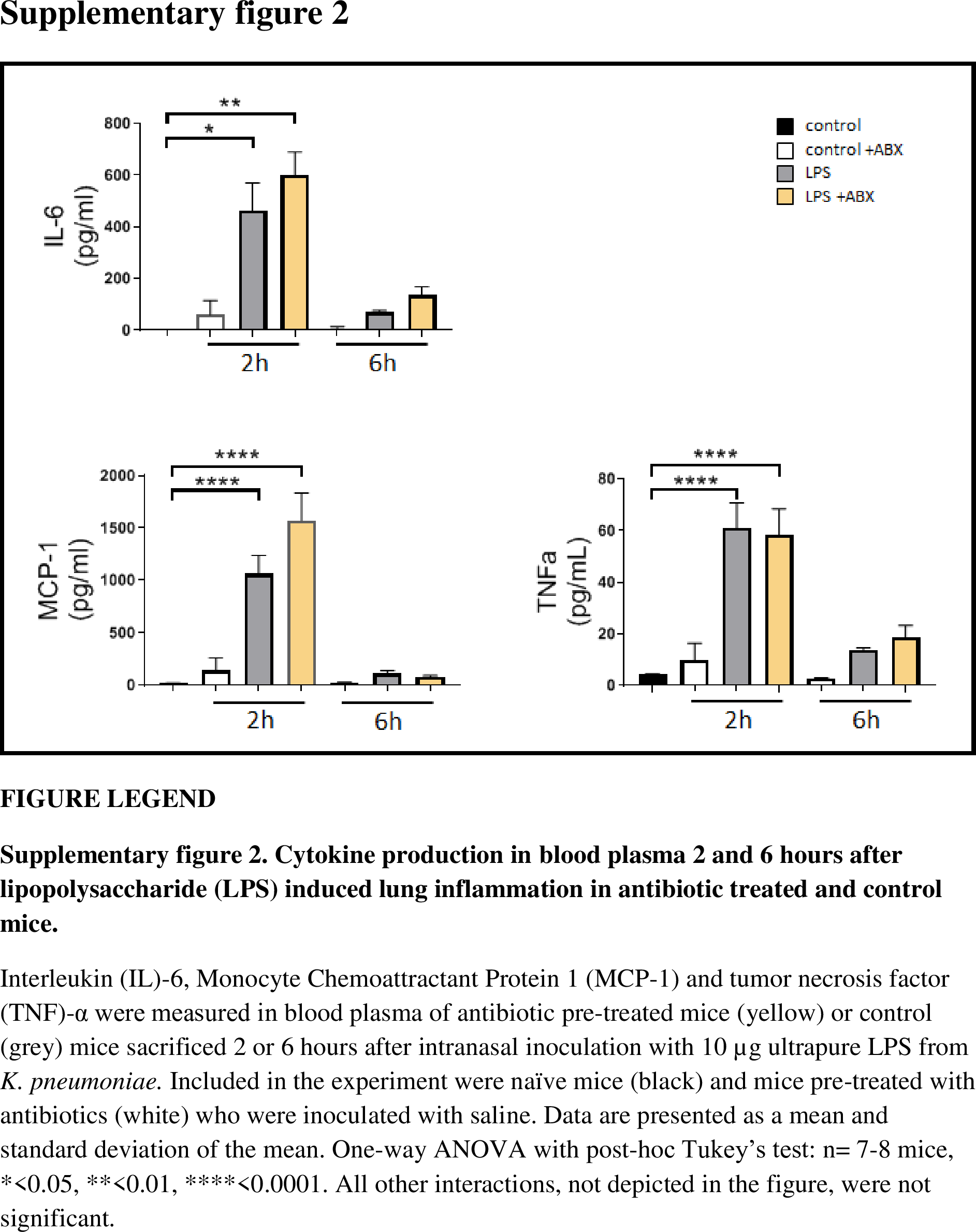

Supplement: S2 Fig — (TIF) [file pone.0241748.s002.tif]

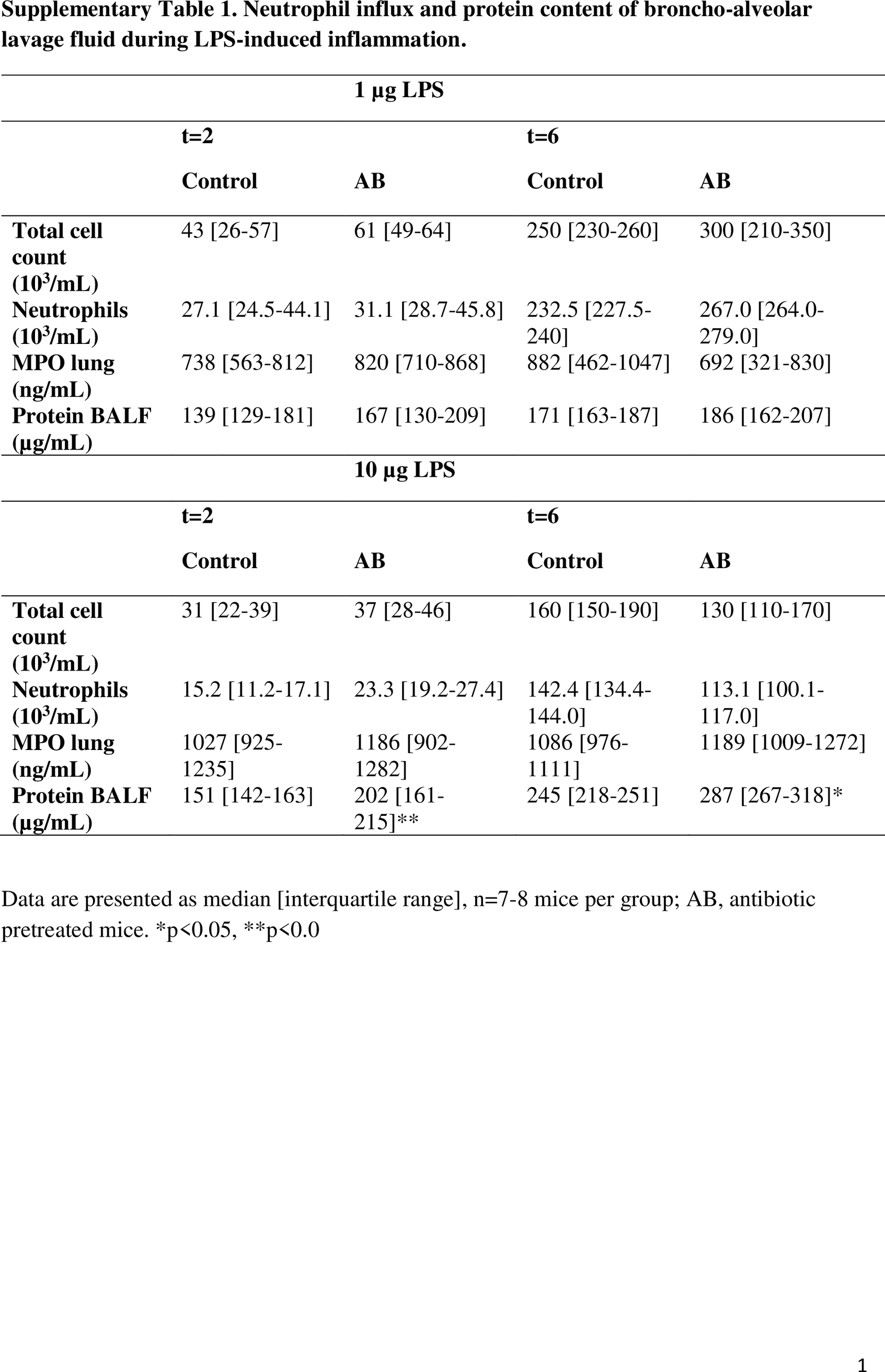

Supplement: S1 Table — (TIF) [file pone.0241748.s003.tif]
